# Retraction: Central Exercise Action Increases the AMPK and mTOR Response to Leptin

**DOI:** 10.1371/journal.pone.0190765

**Published:** 2018-01-02

**Authors:** The PLOS ONE Editors

The editors retract this publication [1] due to concerns over data presented in Figs 2, 5 and 6.

After the publication of this article, concerns were raised that three Western blot images included in this article are highly similar to images used to represent different Western blot experiments elsewhere in this *PLOS ONE* article and in other publications:

- α-JAK2 blot in Fig 2G, α-STAT3 blot in Fig 2H, and panels in other publications.
- α-AMPK blot in Fig 5B and panels in other articles.
- α-ACC blot in Fig 5C, α-4EBP1 blot in Fig 6F, and a panel in another published article.

The authors offered to repeat the experiments, but they were not able to provide the original, uncropped Western blots used to generate the *PLOS ONE* figures.

In light of the concerns about image duplication and data non-availability the *PLOS ONE* editors retract the article.
